# Supplementary material for: Imaging-based assessment of muscles and malnutrition predict prognosis in patients with primary hepatocellular carcinoma
Source: PLoS One. 2025 Apr 24;20(4):e0307458. doi: 10.1371/journal.pone.0307458 (PMC12021244; doi:10.1371/journal.pone.0307458)
Supplement: Attachment — Submitted filename: Response to Reviewers v2.docx [file pone.0307458.s002.docx]

Dear Dr. Nguyen Hai Nam,

Academic Editor

and the reviewers, PLOS ONE

PONE-D-24-25647

Malnutrition as defined by imaging-based sarcopenia and albumin levels predicts prognosis in patients with primary hepatocellular carcinoma

Thank you for your kind review of our manuscript.

We appreciate receiving this opportunity to re-submit our manuscript.

Considering the suggestions, we have written Point by Points. We believe that these changes will alleviate the concerns of the reviewers. We would greatly appreciate if our manuscript could be accepted for publication in PLOS ONE.

Sincerely,

Hitomi Takada, MD.

Gastroenterology and Hepatology Department of Internal Medicine, Faculty of Medicine,

University of Yamanashi,

Yamanashi, Japan

**Point by points**

1. **Since the criteria of sarcopenia, in this manuscript, cannot be met due to the lack of GS measurement data, the Low Muscle Volume and CT Value (LMVV) must be an appropriately alternative option. In this context, the author must edit the manuscript with the limitation of using the word “sarcopenia”.**

Thank you very much for your detailed comments. As you pointed out, LMVV and sarcopenia are different concepts. We stated more carefully that this study is only an examination using LMVV, and excluded sarcopenia from keywords.

We have modified the text as follows. (line 236, page 14)

This study is only an assessment of LMVV, although it is not possible to assess the significance of sarcopenia.

2. **Regarding malnutrition, it’s reasonable to use hypoalbuminemia, Child-Pugh grade B, Subjective Global Assessment (SGA) grade B/C, and Royal Free Hospital Nutrition Prioritizing Tool (RFH-NPT) score >2 as malnutrition factors. However, I strongly suggest to use the combination of these above factors for the establishment malnutrition, instead of separate assessment of each factor.**

Thank you very much for your detailed comments. As you pointed out, malnutrition should be assessed comprehensively, and the text, subtitles, and figures have been revised.

(line 37 page 2)

At baseline, 29% showed LMVV, and 59% met one or more of the malnutrition criteria. No items meeting the criteria of LMVV and malnutrition was observed in 41%, 1 of them was found in 29%, and both were found in 29%.

(line 164 page 8)

Patients with hypoalbuminemia, Child-Pugh grade B, SGA grade B/C, RFH-NPT score >2, and meeting one or more of the malnutrition criteria had a less promising prognosis than those without (p < 0.001) (Figure.2b-f).

(line 176 page 9)

Several independent risk factors, such as age over 78 years, body mass index (BMI) < 18.5, LMVV, malnutrition, alpha-fetoprotein (AFP) levels >20 ng/mL, and des-γ-carboxy prothrombin (DCP) levels >150 mAU/mL, Ammonia > 73 μg/dL, and TNM stages 3/4 were identified for a shorter survival time in patients with primary HCC (Table 2, Figure.2g-l).

(line 188 page 12)

No parameters were found that met the criteria of LMVV and malnutrition, were observed in 295 cases (41%), 1 of them was found in 210 patients (29%), and both were found in 209 cases (29%).

(line 203 page 12)

For liver-related and other types of deaths, the prognosis could be delineating by the number of items answering the criteria of LMVV and malnutrition (Figure.4a, b). Meanwhile, the frequency of liver-related deaths was not influenced by the presence of LMVV alone, while it was connected with malnutrition (Figure.4c, d). The incidence of other types of deaths was influenced by LMVV and malnutrition (Figure.4e, f).

(line 216 page 13)

In this study, we assessed the significance of LMVV and malnutrition in patients with first-diagnosed HCC. Our findings revealed that patients with LMVV and malnutrition at baseline had a lower OS rate than those without, regardless of the HCC stage. LMVV and malnutrition at baseline was identified as an independent prognostic factor for patients with primary HCC.

(line 252 page 14)

We detected that the number of items meeting the criteria of LMVV and malnutrition served as an independent factor for a shorter survival time in all patients and at each stage as well. Meanwhile, the prevalence of liver-related deaths was affected by malnutrition, not LMVV. The incidence of other types of death was associated with LMVV and malnutrition.

(line 265 page 15)

Surprisingly, there are few reports of combined a comprehensive assessment of malnutrition with image-based assessment of skeletal muscle [14, 46].

(line 279 page 15)

The findings of this study emphasize the significance of LMVV and malnutrition as prognostic factors in patients with HCC.

(line 285 page 16)

LMVV and malnutrition are prognostic factors in patients with first-diagnosed primary HCC.

3. **Regarding BCLC staging, since the BCLC stage B group did not show similar results to the other groups, I strongly suggest to remove the patient with BCLC stage B and C; and we just focus on BCLC stage A. Please redo your analysis.**

Thank you very much for your detailed comments. As you pointed out, the failure to stratify prognosis by the combination of LMVV and malnutrition in the BCLC stage B as in other populations, is troubling, and it is an excellent way to exclude these cases and include only BCLC stage A cases.

However, the key findings of this study are that LMVV and malnutrition are important factors across patients with HCC of diverse stages, and that malnutrition and skeletal muscle assessment are important independently of TNM stages. Therefore, we wanted to use the results of all 714 cases and did not restrict this study to BCLC stage A cases and revised the text as follows.

In addition, BCLC staging is a clinically useful classification, but it is a complex classification consisting of items related to HCC staging, liver function and malnutrition. Therefore, it was the author's intention to make a simpler comparison in this study, and the relevance of BCLC staging has been removed.
